# Dissemination of Periodontal Pathogens in the Bloodstream after Periodontal Procedures: A Systematic Review

**DOI:** 10.1371/journal.pone.0098271

**Published:** 2014-05-28

**Authors:** Anna Carolina Ratto Tempestini Horliana, Leandro Chambrone, Adriana Moura Foz, Hilana Paula Carillo Artese, Mariana de Sousa Rabelo, Cláudio Mendes Pannuti, Giuseppe Alexandre Romito

**Affiliations:** Department of Stomatology, School of Dentistry, University of São Paulo, São Paulo, Brazil; University of Toronto, Canada

## Abstract

**Background:**

To date, there is no compilation of evidence-based information associating bacteremia and periodontal procedures. This systematic review aims to assess magnitude, duration, prevalence and nature of bacteremia caused by periodontal procedures.

**Study Design:**

Systematic Review

**Types of Studies Reviewed:**

MEDLINE, EMBASE and LILACS databases were searched in duplicate through August, 2013 without language restriction. Observational studies were included if blood samples were collected before, during or after periodontal procedures of patients with periodontitis. The methodological quality was assessed in duplicate using the modified Newcastle-Ottawa scale (NOS).

**Results:**

Search strategy identified 509 potentially eligible articles and nine were included. Only four studies demonstrated high methodological quality, whereas five were of medium or low methodological quality. The study characteristics were considered too heterogeneous to conduct a meta-analysis. Among 219 analyzed patients, 106 (49.4%) had positive bacteremia. More frequent bacteria were *S. viridans, A. actinomycetemcomitans P. gingivalis*, *M. micros* and species *Streptococcus and Actinomyces*, although identification methods of microbiologic assays were different among studies.

**Clinical Implications:**

Although half of the patients presented positive bacteremia after periodontal procedures, accurate results regarding the magnitude, duration and nature of bacteremia could not be confidentially assessed.

## Introduction

Bacteremia is defined as the transient, intermittent, or continuous presence of bacteria in the bloodstream [1]. It has been argued that the periodontal microbiota in intimate contact with the ulcerated epithelium of gingival sulcus/periodontal pockets is capable of reaching the bloodstream [2], [3].

Molecular techniques have identified DNA sequences from periodontopathogens (e.g. *A. actinomycetemcomitans, P. gingivalis, P. intermedia*) in different organs and systems of the body [4]–[7]. In a recent systematic review [8] authors reported that biofilm accumulation and gingival inflammation increased prevalence of bacteremia after toothbrushing. Moreover, bacteremia is related to the magnitude of tissue trauma, density of bacterial microbiota and inflammation or infection at the site of trauma [9]. The association between periodontal treatment and bacteremia have been reported in several publications [10]–[27] and ranged from 13% [16] to 80.9% [18]; periodontal probing 20% [16] to 43% [24] and periodontal surgery 60% [26]. This wide variation may be attributed to the different employed laboratorial and clinical methods. For detection of bacteremia, culture plates were the most disseminated method [26] but compared with the current techniques it become obsolete. Some authors [13], [17], [28] have reported lysis filtration a very sensitive method that permit to access magnitude whereas conventional broth-based methods are the most commonly used procedure [11], because of the relative convenience and speed of the outcome. However, the principal disadvantage is the diversity of complementary methods for identification.

Recently, the polymerase chain reaction (PCR) has been cited as a more sensitive and specific test that does not depend on bacterial growth, detecting dead organisms degraded by the host immune [16], [21]–[23]. Therefore, diverse microbiological analyses have been performed and do not allow for accurate comparisons to be made, which, in practical terms, can lead to misrepresentative findings. To date, and to our knowledge, there have been no evidence-based compilation studies documenting the association between periodontal procedures and bacteremia. Thus, the purpose of this systematic review was to assess magnitude, duration prevalence and nature of bacteremia induced by periodontal procedures.

## Methods and Materials

### Criteria for considering studies for this review

#### Type of studies

Because our research question was based on dissemination of periodontal bacteria into the bloodstream following periodontal procedures, the most adequate design to answer this question was a systematic review of observational studies. Thus, case series, case-control, cross-sectional and prospective cohort studies were eligible for inclusion. In addition, to reduce potential biases within the review process and to describe a standardized study, this systematic review was prepared in accordance with the MOOSE [30], PRISMA [31] and Check Review [32] checklists (Figure S1).

#### Types of participants and inclusion/exclusion criteria

Minimum sample size of 10 patients healthy patients with aggressive or chronic periodontitis [33] who underwent periodontal procedures: probing pocket depth, prophylaxis, scaling and root planing (hand curettes/scalers or with ultrasonic devices) or periodontal surgery (e.g., open flap debridement, guided tissue regeneration) were included. They must describe at least two blood samples (baseline and one more during/or after periodontal procedures) analyzed by molecular or culture-based methods. Patients submitted to any treatment (i.e. antibiotic treatment), were not included.

#### Outcome measure

A positive diagnosis of oral or non-oral bacteria in the bloodstream following periodontal procedures.

### Search strategy

Search strategies were developed for the MEDLINE, EMBASE and LILACS databases. MesH terms, key words and other free terms were used for searching, and Boolean operators were used to combine searches. Databases were searched through April, 2013, without language restrictions based on the following search strategy developed for MEDLINE (via PubMed): ((((periodontal diseases OR chronic periodontitis OR aggressive periodontitis OR periodontitis OR periodontal pocket)) OR (subgingival curettage OR scaling OR dental scaling OR root planning OR tooth root scaling OR periodontal procedures OR periodontal basic procedures OR periodontal debridement))) AND (bacteremia OR bacteremia OR blood-borne pathogens). Reference lists of previous reviews and potential studies were examined (i.e., hand searching).

### Assessment of validity, data extraction, and methodological quality in included studies

Three review authors did the acquisition of data (A.C.R.T.H., A.M.F. and H.P.C.A.) independently screened titles, abstracts and full texts of the search results. Full text was obtained for all studies that appeared to meet the inclusion criteria or in instances where there was insufficient information from the title or abstract to make a clear decision. Disagreement was resolved by revising it critically for important intellectual content (G.A.R., L.C. and C.M.P.). Data was extracted and recorded in duplicate (A.C.R.T.H., A.M.F., H.P.C.A. and M.S.R.) using specially designed data-extraction forms: citation, publication status, year of publication; study location; characteristics of participants; type of periodontal procedures; method used to assess bacteremia; outcome measures; methodological quality of the study; and source of funding or conflicts of interest. Methodological quality was conducted using The Newcastle–Ottawa scale (NOS scale) [34] modified by others authors [35], [36]. The following topics represented areas of focus: 1) Selection of study groups: sample size calculation; representativeness of the patients with periodontitis; assessment of periodontal conditions; method used to assess bacteremia; calibration of assessors of outcomes; and clear inclusion/exclusion criteria 2) Comparability of patients and management of confounders 3) Outcome of interest: criteria applied to evaluate bacteremia and assessment of outcomes 4) Statistical analysis: appropriateness and unit of analysis. If all criteria of methodological quality were fulfilled within the domains, points (“stars”) were assigned to the respective study. The NOS Scale was adapted for the purpose of this review, and each included study receives a maximum of 12 points. Studies with 9–12 points were considered as high methodological quality, 6–8 points medium and those with <6 points were considered to be of low methodological quality.

### Data synthesis

Data were combined into evidence tables and grouped according to the type of study. A descriptive summary was performed to determine the quantity of the data by further evaluating study variations in terms of the study characteristics and outcomes.

## Results

### Search results and description of included studies

**Figure 1:**
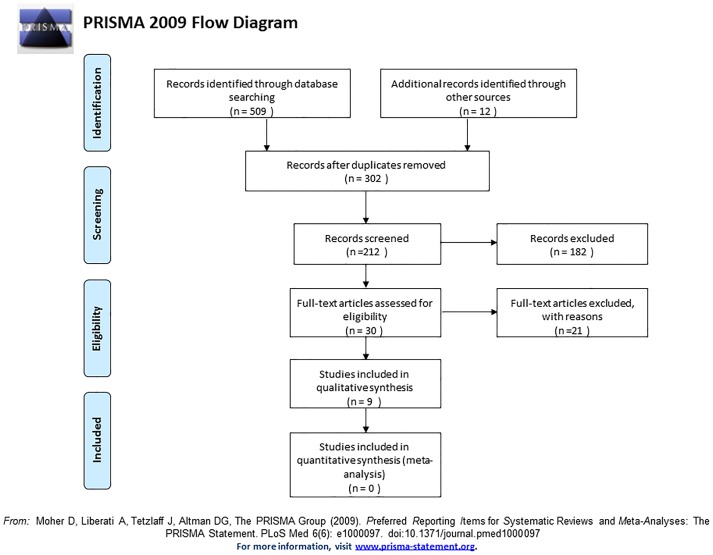
Flow chart of manuscripts screened through the review process.

The search strategy identified 509 potentially eligible articles, of which 479 were excluded after the title and/or the abstract, were reviewed and duplicate removed (Figure 1). Subsequently, 30 of the full texts considered potentially relevant were screened. Of these, 18 [37]–[54] did not fulfill the inclusion criteria (Table 1). The main characteristics of the 9 included studies are listed in the Tables 2, 3 and 4. One study was reported in three different publications [21]–[23], represented by a single study name [18]. In total, 219 patients were assessed within nine cross-sectional studies included in this review, and positive bacteremia were identified in 106 [16]–[20], [24]–[26], [55] of these cases (49.4%).

**Table 1:** Excluded studies and the reason for exclusion.

Study	Reason for exclusion
Lacassin F, Hoen B, Leport C, Selton-Suty C, Delahaye F et al., 1995 [37]	Case control with no bacteremia outcomes
Lieberman MB, 1992 [38]	Case report
Lofthus JE, Waki MY, Jolkovsky D Otomo-Corgel J, Newman MG et al., 1991 [39]	Inclusion of patients undergo periodontal maintenance
Font Buxo J, 1985 [40]	Review
Trivedi DN, 1984 [41]	Review
Wank HA, Levison ME, Rose LF, Cohen DW, 1976 [42]	There was no periodontal treatment
Ewart NP, 1971 [43]	Review
Kraal JH, 1970 [44]	Review
Montanari G, 1957 [45]	not fulfill inclusion criteria of methodology
Bandt, C.L., Korn, N.A., Schaffer, E.M, 1964 [52]	not fulfill inclusion criteria of methodology
Winslow MB, Kobernick SD, 1960 [53]	not fulfill inclusion criteria of methodology
Raetzke P, O'Leary DMD, Miller CH, 1981 [54]	not fulfill inclusion criteria of methodology
Baltch, AL, Shaffer C, Hammer MC 1982 [50]	not fulfill inclusion criteria of methodology
Bayliss R, Clarke C, Oakley et al., 1983 [49]	Case control with no bacteremia outcomes
Conner HD, Haberman S, Collings CK, 1967 [48]	not fulfill inclusion criteria of methodology
Korn NA, EM Schaffer, 1962 [51]	not fulfill inclusion criteria of methodology
Rogosa M, Hamppeg, Nevinta, 1960 [47]	not fulfill inclusion criteria of methodology
Lazansky JP, Robinson L, Rodofsky L., 1949 [46]	not fulfill inclusion criteria of methodology

**Table 2:** Characteristics of the included studies: periodontal probing procedures.

Study	Participants	PD definition	Methods	Outcomes	Conclusion
**Daly and colleagues, 2001 [25]**	40 patients, 21 males and 19 females, mean age 41,8 years, submitted to periodontal probing	Periodontitis group were selected based on radiographic evidence of interproximal alveolar bone loss on a dental panoramic tomography.	BS was collected before and immediately following periodontal probing and were analyzed via aerobic and anaerobic media culture bottles. Gram stained and subculture to appropriate media and isolates identified to genus level.	Microorganisms were identified in the peripheral blood after probing in 40% (8/20) periodontitis patients and in 10% (2/20) gingivitis patients.Frequent MO: *Streptococcus* spp. were the most common isolates in both groups.	“Patients with untreated adult periodontitis are at greater risk of bacteremia due to periodontal probing than patients with gingivitis ”
**Daly and colleagues, 2001 [24]**	30 patients, 15 males and 15 females, mean age 42.7 years, submitted to periodontal probing	Periodontitis group were selected when exhibit radiographic evidence of periodontitis.	BS was collected before and immediately following periodontal probing and were analyzed via aerobic and anaerobic media culture bottles. Gram stained and subculture to appropriate media and isolates identified to genus or species level.	Microorganisms were identified in the peripheral blood before probing in 3 patients (2 of these grew skin commensals and one of these, *Prevotella*). Before (3/30) 10% Immediately after (13/30) 43% Frequent MO: S. v*iridans* were the most common isolates.	“The results indicate that periodontal probing can cause bacteremia in patients with periodontitis ”
**Kinane and colleagues, 2005 [16]**	30 patients, 18 male and 12 female, mean age 42,3 years submitted to periodontal probing	Periodontal disease was defined as having all quadrants with at least one pocket >6 mm, and ≥20 teeth	BS was collected before and immediately after (range 30 s to 1 min) this procedure via blood culture bottles (aerobic/anaerobic) and PCR assay.	Culture methods: Baseline 6% (2/30) Probing (20%) (6/30), PCR analysis: Baseline: (9%) 3/30), Probing 16% (5/30)	“Detectable dental bacteremias induced by periodontal procedures are at a lower level than previously reported”

MO – microorganisms; BS – blood sample; mm – millimeters; PCR – polymerase chain reaction.

Studies of Daly [24], [25] and Kinane [16] are University-based (University of Sydney and United Kingdon respectively)

**Table 3:** Characteristics of the included studies: non-surgical periodontal therapy.

Study	Participants	PD definition	Methods	Outcomes	Conclusion
**Lafaurie and colleagues, 2007 [18]**	42 patients, 25 males and 17 females, mean age 38,15 years, submitted to periodontal treatment	PD was defined as at least 10 pockets with PPD≥7 mm requiring periodontal surgery after SRP	BS was collected before and after SRP (immediately, 15 min, 30 min) and were analyzed via blood culture bottles (anaerobic)	Microorganisms growing under anaerobic conditions were identified in the peripheric blood after SRP in 80.9% (34/42) patients. Evaluation times: T1 - 2,4% (1/42) patients T2 - 73.8% (31/42) patients, T3 - 38% (16/42) patients, T4 - 19% (8/42) patients, Frequent MO *P. gingivalis*, and *M. micros in peripheral blood*	“Scaling and root planning induced bacteremia associated with anaerobic bacteria, especially in patients with periodontal disease”
**Maestre and colleagues, 2008 [20]**	13 patients, 8 males and 5 females, mean age 58.6 years submitted to periodontal treatment	PD was defined as PPD≥4 mm.	BS was collected before and after SRP (1 min) and were analyzed via blood culture bottles (aerobic/anaerobic)	Baseline: 0% (0/13) After treatment 76.9% (10/13) Predominate anaerobic bacteria of the genus *Prevotella*, followed *M. micros and F. nucleatum.*	“Periodontal procedures induce bacteremia and may represent risk of developing systemic complications. The use of antibiotic prophylaxis is crucial for its prevention”.
**Padilla and colleagues, 2007 [21]**	24 patients, 14 males and 10 females, mean age 63,5 years submitted to periodontal treatment	PD was defined as CAL≥5 mm.	BS was collected before and after (5 min.) SRP. Via blood culture bottles (anaerobics)	Baseline 0%. After treatment 29,1% (7/12) of patients with and without atherosclerosis. The most prevalent bacteria in both groups was *A. actinomycetemcomitans*	“Bacteremia occurred in 7/24 patients after SRP. In 4 patients, the same species found in periodontic pockets and blood cultures were selected in atherosclerotic plaques obtained one week after the dental procedure”
**Forner and colleagues, 2006 [17]**	60 patients (20 with periodontitis), 5 males and 15 females, mean age 43,75 years submitted to periodontal treatment	PD was defined as at least 10 sites with PPD>5 mm.	BS was collected before and after (0.5, 10, and 30 min.) SRP. Via lysis filtration (aerobic/anaerobic)	Baseline 0%, 0,5 min: 75% (15/20), 10 min 35% (7/20) , 30 min: 10% (2/20). The isolated bacteria represented a larger variety of species reflecting the increased complexity of the microflora of periodontal pocket	“Patients with periodontitis as compared with healthy individuals and gingivitis patients are at increased risk of experiencing bacteremia in association with scaling” .
**Kinane and colleagues, 2006 [16]**	30 patients, 18 male and 12 female, mean age 42,3 years submitted to periodontal treatmet	Periodontal disease was defined as having all quadrants with at least one pocket >6 mm, and ≥20 teeth	BS was collected before and after (immediately) via blood culture bottles (aerobic/anaerobic) and PCR assay.	Baseline: Culture methods: Baseline - 3% (1/30), SRP - 13% (4/30), PCR analysis Baseline: 13% (4/30), SRP 23% (7/30)	“Detectable dental bacteremias induced by periodontal procedures are at a lower level than previously reported
**Zhang and colleagues, 2013 [55]**	30 patients, 12 males and 18 females, mean age 47 (±9,5) years submitted to periodontal treatment	Periodontal disease was defined as having at list one quadrant (qualified quadrant) with a minimum of five teeth with probing depths ≥5 mm not at the same tooth.	BS was collected before, five minutes 30 seconds and 10 min after periodontal treatment and plated onto chromogenic agar, chocolate agar, and brain heart infusion agar supplemented with vitamin K plates. Any grow was subcultured and identified to at least genus level.	Culture methods: Baseline - 3% (1/30), SRP - 13% (4/30), PCR analysis: Baseline: 13% (4/30), SRP 23% (7/30)	“Detectable dental bacteremias induced by periodontal procedures are at a lower level than previously reported”

SRP – scaling and root planning, PPD Periodontal probing depth, CAL -clinical attachment level, PD – periodontal disease, MO – microorganisms; BS – blood sample; mm – millimeters; PCR – polymerase chain reaction

Lafaurie [18] - University-based (Colombia) This study was supported by the Instituto Colombiano para la Ciencia y la Tecnologia Francisco Jose de Caldas; Maestre Vera [20] - University-based (Spain); Padilla [21] -University-based (Chile); Forner [17] - University-based (Denmark) This study was supported by the Danish Dental Association, Colgate–Palmolive and the Danish Foundation for Mutual Efforts in Dental Care; Kinane [16] - University- based (United Kingdom), Zhang [55] – University-based (Australia).

**Table 4:** Characteristics of the included studies for periodontal surgery.

Study	Participants	PD definition	Methods	Outcomes	Conclusion
**Lineberger, De Marco, 1973 [28]**	21 patients, 8 males and 13 females, mean age 41,8 years, submitted to gingivectomy, flap procedures, and or osteoplasty	Generalized periodontitis group were selected with pocket depth greater than 3 mm in all quadrants	BS was collected before and after the operator had judge maximal trauma from the procedure and were analyzed via aerobic and anaerobic cultured agar pour plates and were characterized on the basis of the morphologic description of the colony, gran staining and biochemical tests.	MO were identified in the peripheral blood after surgical procedures in 60% (6/10) in untreated periodontitis patients and in 40% (4/10) in patients with prior dental prophylaxis and plaque control patients. Frequent MO: Anaerobic/anaeróbic *Diphtheroids*, *S. viridans and Staphylococcus epidermitis* were the most common isolates in both groups.	“A significant incidence of bacteremia occurred during the manipulation of gingival tissues. The use of stimudents, or periodontal surgery in untreated patients and periodontal surgery in treated patients will produce a significant incidence of bacteremia”

MO –microorganisms, BS – blood sample, mm- millimeters, mm – millimeters

Lineberger [28]- University-based (Ohio)

### Bacteremia following periodontal procedures

#### Periodontal probing

Generally, the prevalence of bacteremia after probing was 33,7% (27/80) [16], [24], [25]. Daly et al., (1997) [25], found 43% (13/30) [25] in patients with periodontal disease. In a different sample, Daly et al., (2001) [24] related 40% (8/20) [24] after periodontitis and 10% (2/20) [24] after gingivitis probing. Kinane et al., (2005) [16] found 20% (6/30) for periodontitis patients with culture and 16% (5/30) with PCR methodology. Some of the most common species in pos-probing period were *Micrococcus* [16], *Streptococcus* [24], *Corynebacterium* [24], [25], *Bacteroides* [24], [25], *Desulfomonas* [24], [25], *Peptostreptococcus* [24.25], Gemella [25], *and bacteria S. viridans* [25], [16], *S. milleri* [25] *N. pharynges* [16], *P. intermedia* [16], *A. naeslundii* [16], *H. aphrophilus* [16].

#### Non-surgical periodontal therapy (NSPT)

NSPT led to a total degree of 46% of positive bacteremia [16]–[20], [23], [55] and frequent microorganisms were *A. actinomycetemcomitans* [20], [23], [19] *P. gingivalis* [20], [19], [23], *M. micros* [20], [19], S. viridans [55], *S. sanguis* [16] A. *naeslundii* [16], *S. parasanguis* [16], and species *Actinomyces* [19], [55], [16], *Streptococcus* [17], [16], [20], *Enterococcus* [16], and one isolate of *Candida* [16]. In one study [23] was found 19% of positive bacteremia with nested PCR and 47.6% with anaerobic culture. Some high detected bacteria in subgingival plaque samples (i.e. *T. forsythia, P. intermedia*) were low detected in blood samples [23]. Zhang et al., (2013) [55] found 43.3% of positive bacteremia (13/30) and magnitude of 2±2.0 CFU/mL after NSPT. The highest prevalence was at 5 minutes (10/30) 33.3%. For *S. viridans*, 26.7% (8/30) of samples were positive after NSPT. Kinane et al., (2005) [16] found prevalence of 13% (4/30) for culture and 23% (7/30) for PCR. Forner et al., (2006) [17] found 75% (15/20) and magnitude of 0.78 [0.111–0.67] CFU/mL after 0,5 minutes of NSPT and 35% (7/20) and magnitude of 0.22 [0.11–0.67] CFU/mL in 10 minutes and 10% (2/20) and [0.11–0.11] after 30 minutes. In gingivitis group, all values were minor than in periodontitis. Padilla et al., (2007) [19] found 41,6% (5/12) for healthy and 16% (2/12) for atherosclerosis patients. In Maestre-Vera et al., (2008) [20] study, the prevalence was 76,9% (10/13).

#### Periodontal surgery

Lineberger et al., (1973) [26] found 60% (6/10) of positive bacteremia after diverse surgical procedures: gengivectomy, osteoplasty, and/or flap operation. One of the most prevalent microorganisms was S. v*iridans* (22.9%) for the whole sample.

Overall, the characteristics of the nine included studies (i.e., technique of blood sampling, the point of sampling, time of sampling after periodontal procedure, the type of analysis conducted and treatment provided), were considered too heterogeneous to be combined in a meta-analysis (Table 5). Individual outcomes were reported in Tables 2, 3 and 4. Basically three techniques were used to assess bacteremia: hemoculture, lysis filtration and PCR and all of them detect positive bacteremia, ranging from 13% [16] to 80.9% [18] for NSPT 43 [24] to 20% [16] for probing by culturing and 16% with by PCR analysis [16]. For periodontal surgery [26] a sum of 60% (6/10) positive cases.

**Table 5:** Heterogeneity in methodology and results obtained in different studies selected for this review.

Study	Technique of blood sampling	The point of sampling	Time of sampling	Type of analysis conducted	Type of treatment conducted
**Lafaurie and colleagues, 2007 [18]**	After 1% povidone-iodine disinfection, cannulation was performed by a nurse with 18 GA IV catheter. An injection site adapter was positioned and attached to a sterile multiple sample needle. 5 ml of blood was collected in each sampling	Blood samples were drawn from antecubital vein	T1 = before periodontal procedure; T2 = immediately after periodontal procedure; T3 = 15 min after periodontal procedure; T4 = 30 min after periodontal procedure	blood culture bottles (anaerobic)	Periodontal treatment
**Maestre and colleagues, 2008 [20]**	The technique of blood sample was not described. 20 ml of blood was collected in each sampling	It was not described	T1 = before periodontal procedure; T2 = 1 minute after completion of periodontal treatment	blood culture bottles (aerobic/anaerobic)	Periodontal treatment
**Padilla and colleagues, 2007 [19]**	The blood collection procedure was performed according to the classical aseptic standards. The technique of blood sample was not described. 10 ml of blood was collected in each sampling	It was not described	T1 = before periodontal procedure; T2 = 5 minutes after completion of periodontal treatment	blood culture bottles (anaerobic)	Periodontal treatment
**Forner and colleagues, 2006 [17]**	After 0.5% clorexidina ethanol disinfection, an indwelling catheter was used. This catheter was rinsed with saline after insertion and after obtaining each blood sample. The site of venepuncture was covered by a sterile pad. Two millilitres of blood was discarded before drawing the blood for the bacteremia analysis. 9 ml of blood was collected in each sampling	Blood samples were drawn from antecubital veins	T1 = before periodontal procedure; T2 = 0.5 minutes after completion of periodontal treatment;T3 = 10 minutes after completion of periodontal treatment; T4 = 30 minutes after completion of periodontal treatment	lysis filtration (aerobic/anaerobic)	Periodontal treatment
**Kinane and colleagues, 2006 [16]**	The puncture site was disinfected with isopropyl. Each sample comprised 28 ml of blood using a butterfly (19G) and safety lock blood collection set, 20 ml syringe and vacutainer holder, which were all attached to a Connecta TH three-way stopcock. Two 4.4 ml vacutainer tubes containing ethylenedia- minetetraacetic acid (EDTA) were used for the collection of samples required for PCR analysis.	Blood samples were drawn from veins in antecubital fossa	T1 = before periodontal procedure; T2 = immediately (30 s to 1 min) following periodontal treatment. There are other procedures (toothbrushing and periodontal probing depths)	blood culture bottles (aerobic/anaerobic) and PCR assay	Periodontal treatment
**Zhang and colleagues, 2013 [55]**	Blood samples was obtained using a 25/22 G cannula, which was left in place during each experimental visit to avoid multiple insertions of a needle. The venipuncture technique utilized has been described previously. A 20 ml blood sample was obtained in each sample	Blood samples were drawn from a vein in the antecubital fossa	T1 = before periodontal procedure; T2 = 30 s after periodontal treatment; T3 = 5 min after periodontal treatment; T2 = 10 min. after periodontal treatment	Samples was plated onto chromogenic agar, chocolate agar, and brain heart infusion agar supplemented with vitamin K plates. Any grow was subcultured and identified to at least genus level.	Periodontal treatment
**Lineberger and colleagues, 1973 [26]**	Thirty second application of 2% iodine and then wiped with a sterile 70% alcohol sponge. Each sample consisted of ten milliliters of blood, which was drawn into a vacuntainer using a 20 G needle.	Blood samples were drawn from the median basilica vein in the antecubital fossa to either right or left arm.	T1 = before periodontal procedure;T2 = after the operator had judged maximal trauma from the procedure to have occurred but while the specific procedure was still being carried out.	Were analyzed via aerobic and anaerobic cultured agar pour plates and were characterized on the basis of the morphologic description of the colony, gran staining and biochemical tests	Periodontal surgery (gengivectomy, osteoplasty, and/or flap operation)
**Daly and colleagues, 1997 [25]**	The skin was wiped with polvidone-iodine and 70% alcohol.A 20 ml blood sample was obtained in each sample.	Blood samples were obtained by means of venipuncture.	T1 = before periodontal probing; T2 = immediately following periodontal probing.	Were analyzed via aerobic and anaerobic media culture bottles. Gram stained and subculture to appropriate media and isolates identified to genus or species level.	Periodontal probing
**Daly and colleagues, 2001 [24]**	The skin was wiped with polvidone-iodine and 70% alcohol. A 20 ml blood sample was obtained in each sample.	Blood samples were obtained by means of venipuncture.	T1 = prior periodontal probing; T2 = immediately following periodontal probing	Were analyzed via aerobic and anaerobic media culture bottles. Gram stained and subculture to appropriate media and isolates identified to genus or species level.	Periodontal probing

G- Gauge, IV –Intravascular, T- time, ml-milliliters, PCR –polymerase chain reaction.

### Risk of bias (quality assessment)

**Figure 2:**
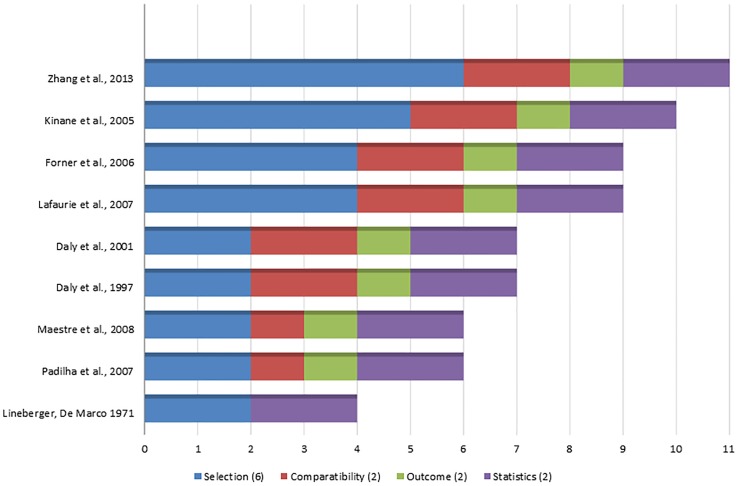
Methodological quality of included observational studies (stars assigned to respective study).

Of the nine included studies, one received an 11-point score (of a total of 12) one a 10-point score, two a 9-point score, two a 7-point score, two a 6-point score, and the last one receive a 4-point score (Figure 2). Thus, 4 studies were considered of high methodological quality [16]–[18], [55], 2 were of medium [24], [25], and 3 were of low methodological quality [19], [20], [26]. In all of the included studies, descriptions of the inclusion/exclusion criteria and unit of analysis (number of patients per group) were considered adequately addressed (i.e., received a star). With exception of one study [26], all of them described standardized methods for the assessment of bacteremia and microbiological analysis. Only one study reported sample size calculations [55], and two [16], [55] presented trained/calibrated assessment of periodontal outcomes, but not a description of a blind examiner. One study [55] reported a blinded assessment for microbiological analysis. Some of the studies had relatively representativeness of patients with periodontitis, [16]–[18], [24], [25], [55] the management of confounders,[16]–[18], [55] and the assessment of periodontal conditions (diagnosis based on full mouth probing measurements or full mouth radiographic evaluation) [16]–[18], [55].

## Discussion

### Summary of the main results

The outcomes of this systematic review reinforce that studies of bacteremia after periodontal procedures in general are poorly designed, and too different in terms of reported methodologies. Despite the heterogeneity related to the time point of blood collection, periodontal diagnosis and microbiological techniques, the prevalence of positive cases of bacteremia after periodontal procedures was 49.4%. More frequent species are not coincident, but in general were *Actinomyces* spp. [19], [55] *Streptococcus* spp [17], [16] and bacteria were *A. actinomycetemcomitans* [20], [23] *P. gingivalis* [20], [19], [23], *M. micros* [20], [19], S. viridans [55]. The duration and magnitude of bacteremia could not be adequately estimated.

### Agreements and disagreements with previous studies

Although a large amount of microorganisms (10^8^ to 10^12^ of Gram-negative bacteria) [57] may be found in untreated periodontal pockets, dental bacteremia is associated with a low magnitude (1–100 CFU/mL) [58]. Therefore, sensitivity of microbiological tests are crucial in bacteremia studies. Traditional broth-based methods (liquid, solid, and biphasic media) are widely used. Some authors [13], [17], [59] considered lysis filtration a more sensitive method compared to hemoculture. Although hemoculture is considered the gold standard [18] and an important diagnostic method to detect alive microorganisms [60], presents some disadvantages. There are two phases to complete the process: detecting bacteria after incubation time and identifying them. The problem really lies with the different methods used for identification. Also, this method can lead to false-negative results because some species (*Prevotella*, *Neisseria*, *Veilonella*) are difficult to cultivate [60], and identify [61], also peculiar oral microbiota requires different culture mediums [2], [17], [59]. This methodology does not detect bacteria degraded by the immune system nor estimate the magnitude of bacteremia [2], [62]. On the other hand, allow rapid response (±10 hours) [62] compared to lysis filtration (2 to 10 days) [13], [17], [62] fundamental in detecting life-threatening septicemia [62].

Culture methods are suitable for detecting alive infective endocarditis microorganisms (i.e S. viridans [55], HANEK - group of fastidious organisms including A. actinomycetemcomitans). As partial solution to these problems, molecular probe-based identification methods (universal 16S rRNA genes or other specific bacterial gene markers) are becoming more popular because of its sensitivity, do not discriminate between live and dead bacteria [16], [23] and identify some uncultivable oral species (i.e. *Prevotella*) [2]. Moreover cannot be utilized in studies for prophylaxis of bacteremia [63] (i.e. antibiotics). A link is being made between *P. gingivalis* and systemic complications [21] including cardiovascular diseases, stroke, lung inflammation, arthritis rheumatoid [64], [21]. This bacterium is capable of binding its fimbriae to endothelial cells [65] and also probably evades the immune system exploiting red blood cells as a transport vehicle rendering it inaccessible to attack by phagocytes [64].

Although it has been identified by culture methods [18], PCR is indicated [21] once red blood cells are lysed as part of the process. Summarizing, it is important to establish bacteria involved in systemic disease, its survival conditions, incubation time, and pathogenicity (alive or dead bacteria) before choose the microbiology test to be used in the study. Four studies using hemoculture detected 73.8% (31/42),[18] 13% (4/30),[16] 76% (10/13) [20] and 29% (7/24) [19] of positive bacteremia. The wide range of prevalence may be attributed to diverse periods of incubations (14 [18], 15 [16], 21 [20] and 35 [19] days) and diversity of time point of blood collection: immediately [16], [18] one [20] and five [19] minutes after NSPT.

Discrepancies must be considered even for the same sample for hemoculture (47.6% – 20/42) [23] and nested PCR (19% - 8/42) [23]. When specific time point was analyzed (immediately after NSPT), hemoculture detect 38% (16/42) [23] whereas nested PCR 21.4% (9/42) [23]. In the same study group [18], 73.8% (31/42) of patients had positive bacteremia immediately after periodontal treatment using hemoculture bottles. PCR would increase the sensitivity and specificity of the detection of periodontal pathogens [16], [23] in this sense, Kinane et al. (2006) [16] found 23% (7/30) of positive bacteremia using PCR and 13% (4/30) with culture method. Some authors [16], [23] used both hemoculture and PCR. It seems techniques would complement each other [23]. Related to molecular methods, newer DNA-DNA hybridization microarrays (bacteremias >10^4^) [63] or 16SrDNA pyrosequencing should be tested [18] in the future, in order to access both more accurate magnitude and diversity of nature of bacteremia. Only one of the included studies uses lysis filtration method [17]. After 30 seconds of NSPT, 75% of patients with periodontitis, 20% gingivitis and 10% with healthy periodontal gum presented bacteremia. These findings seem to be linked to the bacterial load related to the disease; however, no other study using lysis filtration could be included in the review.

Some authors [61] suggested extractions are most likely among dental procedures to cause bacteremia, however periodontal procedures and daily oral activities potentially cause disruption of a larger surface area of inflamed juntional epithelium. Although both procedures have the same access via to the bloodstream, the intensity of trauma is quite different, and also a review of bacteremia in daily oral procedures was performed recently [8]. So we decide to include all periodontal procedures that cause bacteremia via junctional epithelium. Regard to NSPT, some authors [55] suggested that ultrasonic scaler may remove part of bacteria by the flushing action of the water irrigation but others disagree [16] suggesting higher tissue trauma. In included studies, procedures are diverse: full mouth ultrasonic scaling [16], [17] a combination with hand instruments [17], [19], [20] and 10 minutes of scaling and root planning [18] hampering comparison of results.

All the included studies performed baseline blood sampling. In healthy patient that do not realize oral daily activities (at least one hour before the study) [55], [56], and in no contaminated samples (i.e. skin microorganisms) , the results were expected to be null. It is not clear why low-level of transient bacteremia may occur in the absence of therapeutic intervention [18], [16] and clinical relevance of such condition remains unknown. The “pumping action” created by movement of the tooth within the socket [66] (bruxism) should be investigated. In an immune person, the nonspecific host defense (phagocytes and complement) provides the first line protection. In a bacteremic episode, macrophages of the reticuloendotelial system, provide efficient and rapid clearance [16], after blood circulate through the liver [67]. Thus, bacteremia of oral origin is normally fast [58], once repetitive aggressions who's this immune barrier has a strong adapted to attack [16]. Some confounders for bacteremia duration are: heart rate, blood volume and proximity of blood collection to the source of bacteremia [56]. In immunosuppressed patients (i.e. poorly controlled diabetes, leukemia) or treated with immunosuppressing (rheumatoid arthritis, organ transplant) [61], [68] there would be an increased incidence and magnitude of bacteremia [69] and very often attenuated inflammation signs and symptoms. Antibiotic prophylaxis should be individually evaluated. Overall, in questionable cases, balancing of the known risks (i.e. drug reactions, resistant strains) against the possible benefits of antimicrobial regimen (i.e. antibiotics, oral antiseptics), should be evaluated [58], [56].

Plaque accumulation and gingival inflammation significantly increase the prevalence of bacteremia after toothbrushing [8]. There are superficial bacterial effect of mouthrinse in undisturbed dental biofilm [70], [71] and essential oils seems to reduced amount of bacteremia in subjects with gingivitis [15]. They could diminish total load of bacteria before periodontal procedures, especially together with oral hygiene procedures when antibiotic prophylaxis is not indicated. On the other hand, bacterial colonization causing infective endocarditis must be prevented for high susceptible individual undergo invasive procedures [9].

### Quality of the evidence and potential biases in the review process

Heterogeneity of high methodological quality studies [16]–[18], [55] did not allowed comparisons between results, thus meta-analyses of such data may be questionable due to potential bias and the lack of control of confounders [35]. Thus, the most transparent approach was a systematic review of observational studies. Mainly different diagnosis of periodontitis, clinical and microbiological methods, could explain, discrepancies between studies. Also, periodontal treatment should be standardized (i.e. duration of treatment, ultrasonic or hand instruments, scaling or scaling and root planning, full mouth or conventional treatment). Another important issue is the sample size calculations [55], which could underestimated outcomes, once low prevalence of bacteremia require larger samples to achieve statistical differences. Also, a clear description of a blind and calibrated assessment of periodontal outcomes should be cited.

## Conclusions

Almost half of attended patients may present positive bacteremia after periodontal procedures. However, confident results on the magnitude, duration and nature of bacteremia could not be assessed because of the poor design of included studies. Thus, these conditions should be taken into consideration when interpreting the results of this review. Prospective cohort studies and RTCs (comparing different types of procedures) may provide more accurate outcomes on the dissemination of periodontal pathogens into the bloodstream.

## Supporting Information

Figure S1 PRISMA 2009 Checklist
(TIF)
