# Supplementary figures and images for: Dissemination of Periodontal Pathogens in the Bloodstream after Periodontal Procedures: A Systematic Review
Source: PLoS One. 2014 May 28;9(5):e98271. doi: 10.1371/journal.pone.0098271 (PMC4037200; doi:10.1371/journal.pone.0098271)

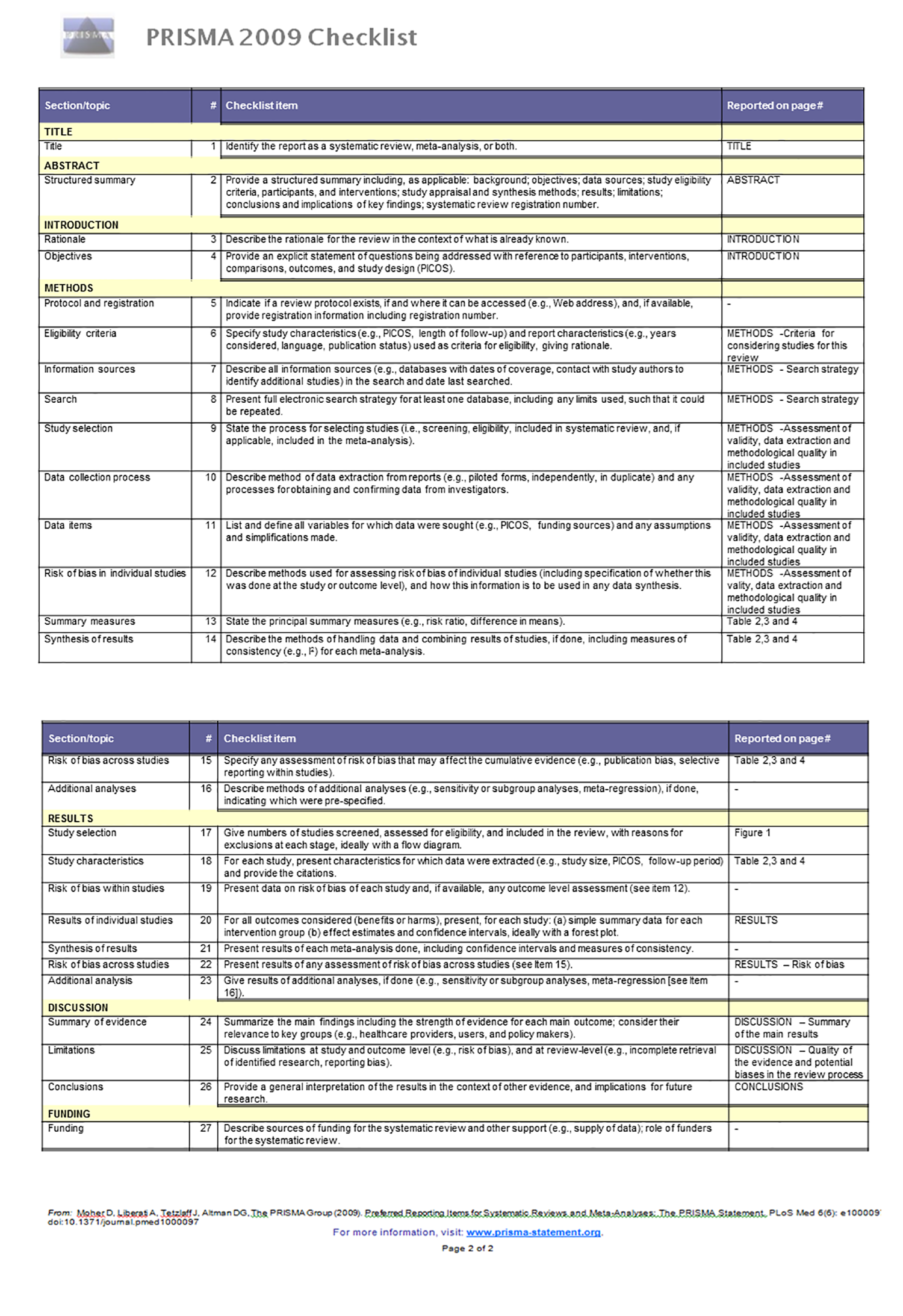

Supplement: Figure S1 — PRISMA 2009 Checklist (TIF) [file pone.0098271.s001.tif]
